# The Institutional Worker

**Published:** 1913-03-01

**Authors:** H. Bond Matron

**Affiliations:** Jersey General Hospital and Dispensary


*To the Editor of* THE HOSPITAL.

### "The Institutional Worker."

*To the Editor of* THE HOSPITAL.

SIR,—I get THE HOSPITAL every week and take a great interest in it as I find it is especially useful since "The Institutional Worker" has been added, which part I hope to see expand as time goes on. I am sure many matrons and heads of hospitals find it just what they want.—Believe me, yours truly, H. BOND, Matron.

Jersey General Hospital and Dispensary,
February 22, 1913.

